# Foliar-applied ethephon enhances the content of anthocyanin of black carrot roots (*Daucus carota ssp. sativus* var. *atrorubens* Alef.)

**DOI:** 10.1186/s12870-017-1021-7

**Published:** 2017-04-04

**Authors:** Gregorio Barba-Espín, Stephan Glied, Christoph Crocoll, Tsaneta Dzhanfezova, Bjarne Joernsgaard, Finn Okkels, Henrik Lütken, Renate Müller

**Affiliations:** 1 Section for Crop Sciences, Department of Plant and Environmental Sciences, Faculty of Science, University of Copenhagen, Hoejbakkegaard Alle 9-13, 2630 Taastrup, Denmark; 2 DynaMo Center, Department of Plant and Environmental Sciences, Faculty of Science, University of Copenhagen, Thorvaldsensvej 40, 1871 Frederiksberg C, Denmark; 3 Natural Colors Division, Chr. Hansen A/S, Agern Allé 24, 2970 Hørsholm, Denmark

**Keywords:** Anthocyanin accumulation, Black carrot, Ethephon, Ethylene, Gene expression

## Abstract

**Background:**

Black carrots (*Daucus carota ssp. sativus* var. *atrorubens* Alef.) constitute a valuable source of anthocyanins, which are used as natural red, blue and purple food colourants. Anthocyanins and phenolic compounds are specialised metabolites, accumulation of which often requires elicitors, which act as molecular signals in plant stress responses. In the present study, ethephon, an ethylene-generating compound was explored as enhancer of anthocyanin and phenolic contents during growth of ‘Deep Purple’ black carrots. The effects of ethephon on several parameters were investigated, and the expression of biosynthetic anthocyanin genes was studied during growth and anthocyanin accumulation.

**Results:**

Roots of ethephon-treated carrot plants exhibited an increase in anthocyanin content of approximately 25%, with values ranging from 2.25 to 3.10 mg g^−1^ fresh weight, compared with values ranging from 1.50 to 1.90 mg g^−1^ fresh weight in untreated roots. The most rapid accumulation rate for anthocyanins, phenolic compounds, soluble solids and dry matter was observed between 10 and 13 weeks after sowing in both untreated and ethephon-treated carrots. The differences in anthocyanin contents between untreated and treated carrots increased for several weeks after the ethephon treatment was terminated. Five cyanidin-based anthocyanin forms were identified, with variable relative abundance values detected during root growth. Overall, the expression of the anthocyanin biosynthetic genes analysed (*PAL1*, *PAL3*, *F3H1*, *DFR1*, *LDOX2*) increased in response to ethephon treatment, as did the expression of the *MYB1* transcription factor, which is associated with activation of the phenylpropanoid pathway under stress conditions. In addition, a correlation was proposed between ethylene and sugar contents and the induction of anthocyanin synthesis.

**Conclusions:**

This study presents a novel method for enhancing anthocyanin content in black carrots. This finding is of economic importance as increased pigment concentration per unit of biomass implies improved profitability parameters in food colour production. We provide new insight into the accumulation patterns of the different cyanidin-based anthocyanins and phenolic compounds during root growth. Moreover, we show that enhanced anthocyanin content in ethephon-treated carrots is accompanied by increased expression of anthocyanin biosynthetic genes.

**Electronic supplementary material:**

The online version of this article (doi:10.1186/s12870-017-1021-7) contains supplementary material, which is available to authorized users.

## Background

In recent years, black carrots (*Daucus carota ssp. sativus* var. *atrorubens* Alef.) have received much attention as a natural source of anthocyanin colourants [1, 2] and many new varieties and old landraces with high anthocyanin content are now being cultivated [3]. Anthocyanins are widely occurring water-soluble pigments belonging to the flavonoid group of phenolic compounds. To date, over 600 different anthocyanins have been identified from plant sources comprising six common anthocyanidin aglycones (pelargonidin, cyanidin, peonidin, delphinidin, petunidin and malvidin) and numerous glycosylated and acylated compounds [1, 4]. In mature black carrot taproots, acylated cyanidin glycosides represent the major fraction of anthocyanin compounds [5–7], although trace amounts of peonidin- or pelargonidin-based anthocyanins have been identified in some cultivars [2].

Due to both increasingly rigorous legal restrictions and consumer concerns, there is increasing demand for natural food colourants that can be used as substitutes for synthetic colours [8, 9]. Anthocyanins provide bright red, blue and purple food colours [10, 11], and represent excellent replacements for artificial colours due to their physico-chemical properties (high pH, light, and heat stability). Acylation has crucial effects on anthocyanin colour and stability [12, 13]. Moreover, anthocyanins possess putative health benefits as dietary antioxidants [14]. In addition to anthocyanin as the predominant polyphenol, black carrots contain large amounts of other phenolic compounds, such as hydroxycinnamates and caffeic acid [5].

Phenolic compounds are regarded as both specialised metabolites and antioxidants [12, 15]. The accumulation of specialised metabolites often requires elicitors, which act as molecular signals in plant stress responses [16, 17]. To our knowledge, there are no reports describing enhancement of anthocyanin content through the use of elicitors in black carrot taproots. Anthocyanin biosynthesis has been extensively studied in the fruit, leaves and flowers of several plants species [18–21]. In black carrots, most of the structural genes participating in the anthocyanin biosynthesis pathway have been identified [22–24]. However, the mechanism by which this pathway is regulated during root growth remains unknown.

In the present study, we investigated the potential function of ethephon, an ethylene-generating compound, as an elicitor of anthocyanin content in field-grown black carrots. Anthocyanin composition was monitored during root growth to determine the onset of the elicitation by ethephon and its effect on the accumulation of the different anthocyanin forms. In parallel, the concentration of phenolic compounds was monitored. We also investigated the effects of ethephon on the dry weight and sugar content, and the expression patterns of certain anthocyanin biosynthetic genes (representing the early, middle, and later stages of the biosynthetic pathway) during growth and anthocyanin accumulation. Beyond the implications that increased anthocyanin content per unit of biomass have for colour production, this research provides new insights into the regulation of biosynthesis and accumulation patterns of the different cyanidin-based anthocyanins during root growth.

## Methods

### Plant material, field conditions and elicitor treatment

Carrot seeds ‘Deep Purple’ F1 were provided by Bejo Seeds, Inc. (Oceano, CA, USA). Three-row plots were arranged in randomised block designs with three replicates. In 2014 and 2015, trials were conducted at the University of Copenhagen, Hoejbakkegaard (Denmark), where foliar applications of ethephon (CERONE® brand ETHEPHON, Bayer Crop Science, Leverkusen, Germany) were performed with a CO_2_ backpack spaced 50 cm apart. In 2016, trials were conducted in at the experimental station at the University of Madeira (Funchal, Portugal), where ethephon was applied using manual spraying equipment designed for agricultural use. The use of ethephon was performed in accordance with its labelling and the protection standard. Ethephon application began 6 weeks after sowing and continued every 3 weeks, with a total of six applications. Plants were cultivated in loamy soil using techniques recommended for cultivation and plant protection in carrot crop production. In addition to natural precipitation, carrots were irrigated as required throughout the growing period.

Plants were cultivated in two types of plot arrangements. Small plots consisted of 4.5 m-long rows, with a single harvest from the middle part of each row performed at 21 weeks after sowing. Large plots consisted of 12 m-long rows, with several harvests from separate row segments performed during growth (7, 10, 13, 16, 19, 22, 25, 28, 29 and 35 weeks after sowing). The ethephon concentrations, sowing dates, field location and harvest dates of the different trials are specified in Table 1.

**Table 1:** Sowing dates, harvest dates, ethephon concentrations and locations of the different field trials conducted during the 2014, 2015 and 2016 growing seasons. DK: Denmark; PT: Portugal

Harvest	Trial sowing date	Trial harvest date(s)	Ethephon (g ha^−1^)	Field location
Single	19/05/2014	12/10/2014	0, 120 and 360	DK
	18/05/2015	12/10/2015	0, 360 and 720	DK
	25/05/2015	19/10/2015	0, 360 and 720	DK
	29/02/2016	22/07/2016	0 and 360	PT
	06/03/2016	29/07/2016	0 and 360	PT
Multiple	18/05/2015	2015: 29/06; 20/07; 10/08; 31/08; 21/09; 13/10; 02/11; 23/11; 30/11. 2016: 19/01	0 and 360	DK

### Sample preparation

After harvesting, 20 whole carrot taproots per plot (biological replicates) were washed and split lengthwise. Of these, 20 halves were ground to a powder under liquid nitrogen before storage at −80 °C for further gene expression analyses. The complementary 20 halves were coarsely ground and homogenised in a 3% sulfuric acid solution (1/1, *w*/w), using a Waring® two-speed commercial blender (VWR - Bie & Berntsen, Herlev, Denmark). The resulting fine homogenate was subsequently mixed with demineralised water (1/2, *w*/w) [or 70% ethanol (1/2, *w*/w) for the analysis of total phenolic content] and vortexed. After incubation for 1 h at room temperature, the sample was centrifuged for 20 min at 4500 rpm, and the supernatant (extract) was utilised for further analyses.

### Determination of total monomeric anthocyanin content (TMA)

TMA was measured spectrophotometrically according to the pH differential method [11], with slight modifications. The carrot extract was diluted by adding 20 volumes of 0.2 M KCl-HCl (pH 1), and the absorption was measured between 350 and 700 nm using a UV-visible spectrophotometer (Thermo Scientific Evolution™ 220, Waltham, MA, USA). The TMA was expressed as cyanidin-3-glucoside equivalents.

### Determination of total phenolic content (TPC)

TPC was measured using the Folin–Ciocalteau method [25]. In brief, 100 μL of carrot extract were mixed with 0.5 mL of Folin–Ciocalteau reagent, followed by the addition of 1 mL of 20% (*w*/*v*) sodium carbonate. After incubation for 2 h in darkness, the absorbance was measured at 760 nm using a UV-visible spectrophotometer (Thermo Scientific Evolution™ 220. The TPC was deduced from the calibration curve, and the results were expressed as mg of gallic acid equivalent (GAE) per g of fresh weight (FW).

### Determination of dry weight (DW) and soluble solids content (SSC)

SSC was calculated with a manual refractometer (Refracto 30PX/GS Mettler-Toledo Inc., OH, USA) operating in the 0% to 85% Brix range. The carrot extract was filtered through 0.45 μm membrane filters, and Brix measurements were performed using 1 mL of the filtrate.

After samples were dried to a constant weight at 100 °C for 24 h, DW was determined based on the difference in mass between the fresh and dry samples. SSC was then expressed as a percentage of the dry matter.

### High performance liquid chromatography-diode array detection (HPLC-DAD)

HPLC-DAD analysis was performed to identify and relatively quantify individual anthocyanins in carrot samples. Carrot extracts were filtered through a 0.45 μm membrane filter prior to analyses. Samples were analysed on an Elite Lachrom HPLC system coupled with a photodiode array detector (L2450), pump, and autosampler (L2200) controlled by EZ Chrom Elite software. The sample was injected using a Lichrosorb RP-18 column (5 μm, 4.6 mm × 250 m) (Alltech, Copenhagen, Denmark). Spectroscopic data were recorded in the wavelength range from 250 nm to 700 nm during gradient elution using a mobile phase composed of (A) water/formic acid/acetonitrile (87/10/3, *v*/v/v) and (B) water/formic acid/acetonitrile (40/10/50; *v*/v/v) at a flow rate of 0.8 mL min^−1^. The analysis conditions were as follows: 0 min, 6% B; 20 min, 20% B; 35 min, 40% B; 40 min, 60% B; and 45 min, 90% B; followed by a 10-min equilibration period. The anthocyanins were identified by comparison of retention times and UV/vis spectroscopic data to known, previously reported data. The relative abundance of each form identified was calculated by integration of the corresponding chromatogram peak area.

### Liquid chromatography coupled to (quadrupole) time-of-flight mass spectrometry (LC-MS/Q-TOF)

To complement the HPLC-DAD analyses, filtered carrot extracts were subjected to LC-MS/Q-TOF analysis to identify and characterise anthocyanins based on accurate molecular mass and fragmentation patterns. Chromatography was performed on a Dionex UltiMate® 3000 Quaternary Rapid Separation UHPLC+ focused system (Thermo Fisher Scientific, Germering, Germany). Separation was performed by gradient elution on a Kinetex 1.7u XB-C18 column (1.7 μm, 100 Å, 100 × 2.1 mm; Phenomenex, Torrance, CA, USA) using a mobile phase composed of (A) water/formic acid (99.95/0.05, *v*/v) and (B) acetonitrile/formic acid (99.95/0.05, *v*/v) at a flow rate of 300 μL min^−1^. The analysis conditions were as follows: 0.0 min, 2% B; 1.0 min, 2% B; 20.0 min, 40% B; 25.0 min, 100% B; 26.9 min, 100% B; 27.0 min, 2% B; and 30.0 min, 2% B. The column temperature was maintained at 30 °C. Four wavelengths (280 nm, 320 nm, 360 nm and 520 nm) were monitored using a UV-VIS detector. The liquid chromatography was coupled to a Compact micrOTOF-Q mass spectrometer (Bruker, Bremen, Germany) equipped with an electrospray ion source (ESI) operated in positive ionisation mode. The ion spray voltage was maintained at +4500 V. Dry temperature was set to 200 °C and dry gas flow was set to 8 L min^−1^. Nebulising gas was set to 2.5 bar and collision energy to 15 eV. Nitrogen was used as the inert gas, nebulising gas and collision gas. AutoMSMS mode was used to obtain MS and MS/MS spectra of the three most abundant ions. The *m/z* range was set to 50–1400. All files were automatically calibrated based on Na^+^-formate clusters injected at the beginning of each run. Quality control samples (QC), consisting of a pool of equivalent aliquots of individual samples, were used to monitor technical variation.

### RNA isolation and cDNA synthesis

Total RNA was extracted from ground carrots with RNeasy® Plant Mini Kits (Qiagen, Hilden, Germany) and subsequently treated with DNase I Amplification Grade (Sigma–Aldrich, MO, USA) to eliminate DNA contamination, according to the manufacturers’ instructions. RNA quality and quantity were evaluated using agarose gel electrophoresis and a NanoDrop™ 1000 Spectrophotometer (Thermo Fisher Scientific, MA, USA), respectively. Total RNA (2 μg) from each sample was used to synthesise cDNA in a 20 μl reaction volume using the cDNA iScript™ Synthesis Kit from Bio-Rad (CA, USA) according to the manufacturer’s instructions.

### Real-time quantitative PCR

Real-time quantitative PCR analysis was performed in a 20 μL reaction volume, using 5 μL cDNA (diluted 1:100) as a template with 10 μL of 1× Power SYBR® green PCR master mix (Applied Biosystems, Warrington, UK) and 0.4 μM of each primer. Threshold cycles (Ct) for target gene expression were standardised to *DcActin*2 [26] Ct (ΔCt). The relative quantification of target gene expression between the different harvests was determined according to the 2^(−ΔΔCt) method as described previously [27]. Nucleotide sequences of primer pairs specific for each gene are provided in Additional file 1: Table S1. Primers specific for *PAL3*, *F3H1*, *DFR1* and *LDOX2* genes have been reported previously [24]. Primers specific for *Actin2*, *EIN3*, *MYB1* and *PAL1* genes were designed using Primer3 online software (http://bioinfo.ut.ee/primer3-0.4.0/primer3/). Primer efficiency was assessed by plotting the cycle threshold value (Ct) at each concentration against the logarithm of the fold-dilution of the sample. Experiments were conducted in duplicate with biological triplicates.

### Statistical analyses

All analyses were performed with three biological replicates. Data were subjected to statistical analysis using the R 3.0.0 statistical package (MA, USA). Data from the accumulation curves were analysed with the lmer function of the lme4 R package. This function is used for linear mixed model including the block design as a random effect. Treatments were compared using one- or two-way analysis of variance (ANOVA) followed by a Tukey post-hoc test. *p* ≤ 0.05 was considered to indicate statistical significance.

## Results

### Effect of different ethephon concentrations on anthocyanin accumulation

**Fig. 1:**
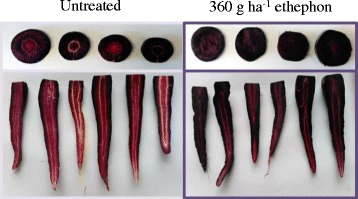
Cross and longitudinal sections of roots of untreated and 360 g ha^−1^ ethephon-treated black carrot plants at 21-weeks after sowing

In the present study, the function of ethylene as a pre-harvest elicitor of anthocyanin pigments was investigated in black carrots following foliar spray with ethephon. First, the effect of ethephon on TMA was analysed in the roots of 21 week-old plants. Overall, ethephon-treated plants displayed intensive purple pigmentation throughout the whole root. In contrast, the pigmentation was less intense in untreated plant roots, being visible both in cross and longitudinal sections (Fig. 1). The mean root TMA of treated plants ranged from 2.25 to 3.10 mg g^−1^ FW, representing an increase of 25% compared with the the values of untreated plants (1.50–1.90 mg g^−1^ FW) (Table 2). However, there were no significant differences in root TMA between the groups treated with 120, 360 and 720 g ha^−1^ ethephon. Roots from carrots sown on 25 May, 2015 displayed the highest anthocyanin concentration following ethephon treatment, reaching 3.10 ± 0.06 mg g^−1^ FW, which was 1.17 mg g^−1^ FW higher than that of the respective control (1.83 ± 0.22 mg g^−1^ FW). Relative to the dry weight, the mean root TMA of treated plants increased by an average of 60%, due to the lower DW of treated carrots. In addition, the roots of ethephon-treated plants sown on the 29 February, 2016 and 6 March, 2016 showed lower SSC. This difference was more pronounced, reaching 13%, in carrots sown on 29 February, 2016 (15.1 and 12.3 °Brix for untreated and treated plants, respectively) (Table 2). In contrast, ethephon applications had no effects on root FW through either growing season (Table 2) or on carrot production in tonnes per hectare (data not shown).

**Table 2:** Total monomeric anthocyanin content (TMA) and yield parameters in roots of ethephon-treated carrot plants in trials harvested at a single time-point (21 weeks after sowing)

Trial sowing date	Ethephon (g ha^−1^)	TMA		Root weight (g)	SSC	DW
		mg g^−1^ FW	mg g^−1^ DW			
19/05/2014	0	1.50 ± 0.33^b^	14.73 ± 0.77^b^	82.04 ± 3.75^a^	14.01 ± 0.88^a^	10.21 ± 0.33^a^
	120	2.48 ± 0.17^a^	28.47 ± 1.28^a^	81.16 ± 6.19^a^	13.03 ± 0.41^a^	9.78 ± 0.26^a^
	360	2.25 ± 0.08^a^	23.88 ± 2.43^a^	82.45 ± 4.57^a^	12.95 ± 0.37^a^	9.43 ± 0.44^a^
18/05/2015	0	1.90 ± 0.09^b^	19.30 ± 0.85^b^	89.06 ± 5.21^a^	13.33 ± 0.08^a^	9.86 ± 0.32^a^
	360	2.63 ± 0.09^a^	29.90 ± 1.23^a^	91.37 ± 3.90^a^	12.33 ± 0.58^a^	9.01 ± 0.16^a^
	720	2.60 ± 0.20^a^	28.37 ± 2.64^a^	88.33 ± 2.14^a^	12.48 ± 0.30^a^	9.22 ± 0.37^a^
25/05/2015	0	1.83 ± 0.22^b^	18.30 ± 2.27^b^	86.14 ± 2.13^a^	13.81 ± 0.25^a^	10.02 ± 0.28^a^
	360	3.10 ± 0.06^a^	34.12 ± 0.65^a^	87.22 ± 4.16^a^	12.46 ± 0.11^a^	9.67 ± 0.23^a^
	720	2.95 ± 0.10^a^	31.50 ± 1.75^a^	84.15 ± 1.75^a^	12.41 ± 0.39^a^	9.39 ± 0.73^a^
29/02/2016	0	2.13 ± 0.19^a^	14.58 ± 0.89^b^	91.52 ± 3.93^a^	15.08 ± 0.81^b^	13.72 ± 0.44^b^
	360	2.52 ± 0.24^a^	21.10 ± 1.98^a^	115.42 ± 13.78^a^	12.33 ± 0.53^a^	11.94 ± 0.09^a^
6/03/2016	0	1.90 ± 0.18^b^	14.29 ± 1.11^b^	81.31 ± 5.84^a^	13.68 ± 0.64^b^	13.32 ± 0.18^b^
	360	2.44 ± 0.19^a^	22.36 ± 2.23^a^	96.59 ± 15.00^a^	11.7 ± 0.52^a^	10.92 ± 0.35^a^

*SSC* soluble solids content *FW* fresh weight, *DW* dry weight. Different superscript letters (“a” and “b”) indicate statistical significance according to Tukey’s test (*p* ≤ 0.05). When only two groups existed (trial sowing dates 29/02/2016 and 06/03/2016), *t*-test was performed to compare means

### Anthocyanin accumulation during root growth

**Fig. 2:**
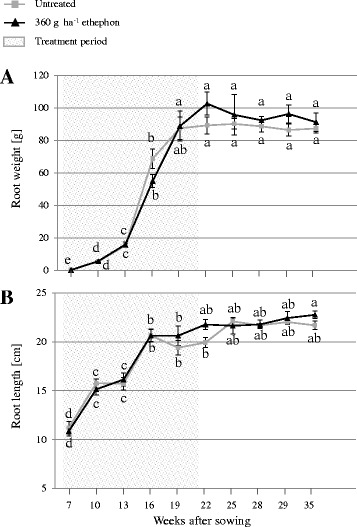
(**a**) Root weight and (**b**) length monitored in untreated and 360 g ha^−1^ ethephon-treated black carrot plants (7–35 weeks after sowing). Different letters indicate statistical significance according to Tukey’s test (*p* ≤ 0.05). Data represent the mean ± SE, *n* = 3

**Fig. 3:**
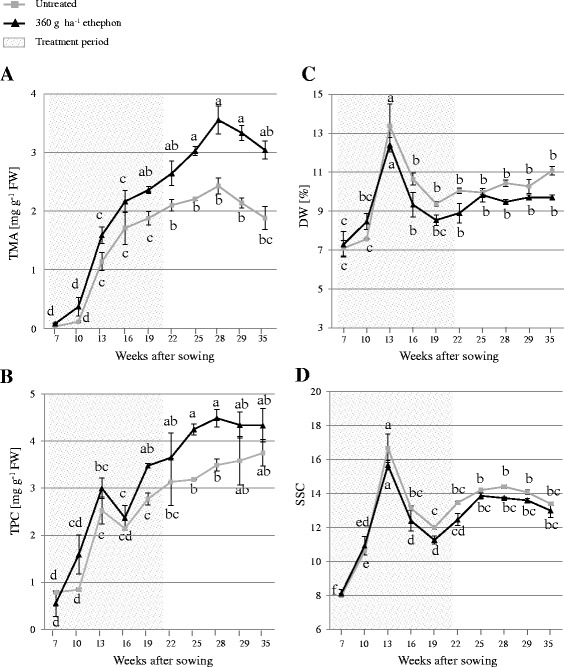
(**a**) Total monomeric anthocyanin content (TMA), (**b**) total phenolic content (TPC), (**c**) dry weight (DW), and (**d**) soluble solids content (SSC) monitored in roots of untreated and 360 g ha^−1^ ethephon-treated black carrot plants (7–35 weeks after sowing). Different letters indicate statistical significance according to Tukey’s test (*p* ≤ 0.05). Data represent the mean ± SE, *n* = 3

The effect of 360 g ha^−1^ ethephon on TMA, TPC, SSC, DW and root size was evaluated during the whole growing period, from 26 June 2015 to 19 January 2016 (7, 10, 13, 16, 19, 22, 25, 28, 29 and 35 weeks after sowing) (Figs. 2 and 3). There were no significant differences in root weight and length between untreated and treated carrots at each harvest point (Fig. 2). However, compared with untreated plants, the mean root anthocyanin concentration was higher in treated plants at every harvest point. Differences between the mean root anthocyanin concentrations of untreated and treated plants continued to increase until 7 weeks after the last ethephon treatment, reaching 3.56 ± 0.24 and 2.43 ± 0.14 mg g^−1^ FW in treated and untreated plants, respectively, at 28 weeks after sowing (Fig. 3a). TPC also increased with root growth over time (Fig. 3b). Based on the difference between the TMA and TPC, the percentage of anthocyanin in the TPC in the roots of both untreated and treated plants ranged from approximately 10% at the early harvest points to nearly 75% at the late harvests (data not shown). Overall, DW and SSC were reduced during growth following ethephon treatment. For both treated and untreated carrots, DW and SSC accumulation curves exhibited a marked peak 13 weeks after sowing, followed by a pronounced decrease and a plateau phase (Fig. 3c and d). Compared with the untreated group, significantly lower SSC was achieved 19 weeks after sowing following ethephon treatment.

### Identification and characterisation of anthocyanins

**Fig. 4:**
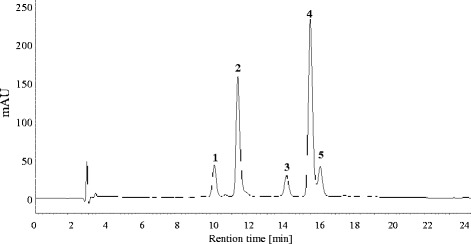
Typical HPLC-chromatogram of anthocyanins in black carrot roots recorded at 520 nm. Peak identification (1–5) is shown in Table 3

Identification of anthocyanins was performed based on the UV-visible spectra, retention time, as well as accurate mass and fragmentation patterns determined by LC-MS, in addition to comparisons with reported data from black carrot (Fig. 4; Table 3). The anthocyanins detected were cyanidin-based forms: cyanidin 3-xylosyl(glucosyl)galactoside (peak 1), cyanidin 3-xylosylgalactoside (peak 2), cyanidin 3-xylosyl(glucosyl)galactoside acylated with sinapic acid (peak 3), cyanidin 3-xylosyl(glucosyl)galactoside acylated with ferulic acid (peak 4), and cyanidin 3-xylosyl(glucosyl)galactoside acylated with coumaric acid (peak 5). However, peonidin 3-xylosylgalactoside, which was detected in trace amounts in previous studies [2, 28], was not detected in the current study. MS/MS analyses of anthocyanins containing cyanidin aglycone yields a characteristic fragment at *m/z* 287, corresponding to the neutral loss of the hexose moiety; all peaks provided this fragment in our study (Table 3). The prevalent peak 4 (*m/z* 919.2509) in both untreated and elicited carrots corresponded to cyanidin 3-xylosyl(feruloylglucosyl)galactoside, which is the major acylated anthocyanin in carrot roots [28].

**Table 3:** Identification of the anthocyanins in the black carrot extracts by LC-MS/Q-TOF in positive ionisation mode. The anthocyanin profile is consistent with those previously published for ‘Deep Purple’ [2, 28, 37–39]. Theoretical and measured molecular masses are shown for the positive ion [M]^+^ and the aglycon

Peak	Retention time [min]	Molecular formula	*m/z* [M]^+^		*m/z* Aglycon	Anthocyanin identity
			(Theoretical)	(Measured)		
1	7.4	C_32_H_39_O_20_	743.2029	743.2032	287.0543	cyanidin 3-xylosyl(glucosyl)galactoside
2	7.8	C_26_H_29_O_15_	581.1501	581.1516	287.0549	cyanidin 3-xylosylgalactoside
3	8.7	C_43_H_49_O_24_	949.2608	949.2600	287.0546	cyanidin 3-xylosyl(sinapoylglucosyl)galactoside
4	9	C_42_H_47_O_23_	919.2502	919.2509	287.0540	cyanidin 3-xylosyl(feruloylglucosyl)galactoside
5	10.2	C_41_H_45_O_22_	889.2397	889.2404	287.0543	cyanidin 3-xylosyl(coumaroylglucosyl)galactoside

**Fig. 5:**
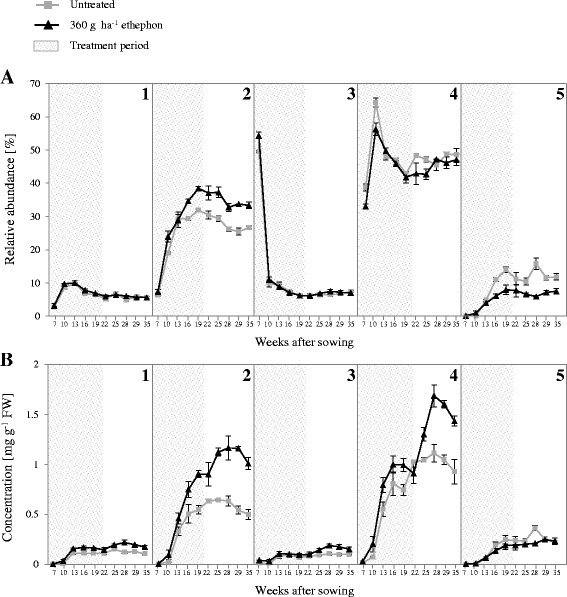
Anthocyanin composition monitored in roots of untreated and 360 g ha^−1^ ethephon-treated black carrot plants (7–35 weeks after sowing). (**a**) Relative abundance (%) of each anthocyanin form calculated from integration of the corresponding chromatogram peak area. (**b**) Concentration of each anthocyanin form estimated based on total anthocyanin content (TMA) and relative abundance of each peak. Peak identification (1–5) is shown in Table 3. Data represent the mean ± SE, *n* = 3

The relative abundance of the five cyanidin-based anthocyanin forms was monitored during root growth, through calculation of the percentage peak area from HPLC chromatograms (Fig. 5a). Based on this value and the TMA, the concentration of each anthocyanin form was also estimated at each harvest point (Fig. 5b). The five anthocyanin peaks were detected at all harvest points analysed, with the exception of the form acylated with coumaric acid, which was first detected 10 weeks after sowing (Fig. 5). Approximately 86% of the total anthocyanins comprised acylated forms (peaks 3–5) at 7 weeks after sowing, mainly due to the high proportion (50%) of cyanidin 3-xylosyl(sinapoylglucosyl)galactoside at this time-point. Thereafter, the abundance of the pool of acylated anthocyanins (peaks 3–5) stabilised at approximately 58% of the total anthocyanins (Fig. 5a); for example, at 28 weeks after sowing (when anthocyanin concentrations reached a maximum), 2.10 mg g^−1^ of the 3.56 mg g^−1^ FW of the total root anthocyanins in the treated plants represented acylated forms (peaks 3–5; Fig. 5b). Overall, both untreated and elicitor-treated carrots displayed similar relative abundances of the different anthocyanins over time.

### Relative expression of anthocyanin biosynthesis-related genes

**Fig. 6:**
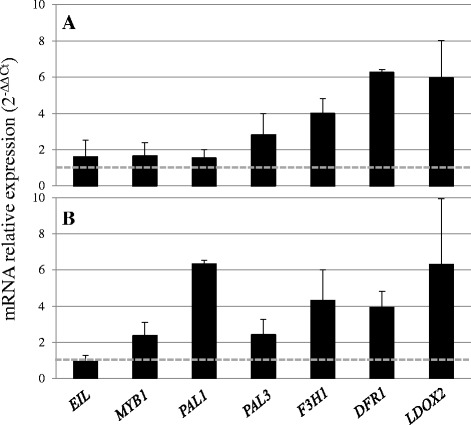
Fold changes in target gene expression in roots of 360 g ha^−1^ ethephon-treated black carrot plants relative to untreated plants (dashed horizontal line) at 22 (**a**) and 25 (**b**) weeks after sowing. Threshold cycles (Ct) for target genes are standardised to the *DcActin2* Ct (ΔCt). The relative expression of target genes in the different harvests is determined according to the 2^(−ΔΔCt) method. Expression levels of target genes in untreated carrots were assigned an arbitrary value of 1. Data represent mean ± SE, *n* = 3

The expression of five anthocyanin structural genes [phenylalanine ammonia-lyase (*PAL1* and *PAL3*), flavanone 3-hydroxylase (*F3H1*), dihydroflavonol 4-reductase (*DFR1*), and leucoanthocyanidin dioxygenase (*LDOX2*)], *EIN3-like* (*EIL*), and *MYB1* genes was quantified in untreated and 360 g ha^−1^ ethephon-treated carrots, at 22 weeks (Fig. 6a) and 25 weeks (Fig. 6b) after sowing. Transcripts of four of these genes (*PAL1*, *F3H1*, *DFR1, LDOX2*) and, to a lesser extent, *PAL3*, accumulated to high levels in treated carrots (2- to 6-fold compared with the levels in untreated plants). Remarkably, *PAL1* and *MYB1* transcript levels were increased in the roots of 25-week-old plants (2.3- and 6.3-fold change, respectively, compared to untreated plants). In contrast, *EIL* was constitutively expressed in both treated and untreated plants (Fig. 6).

## Discussion

### Ethephon as an anthocyanin elicitor

In this study, we showed that foliar-application of ethephon elicited anthocyanin accumulation in the roots of black carrot plants (Table 2). Collectively, our results demonstrate that ethephon treatment can be used to increased anthocyanin content in roots grown in temperate (Denmark) and a subtropical (Madeira) regions. Thus, our findings validate the use of ethephon as anthocyanin elicitor in black carrot roots cultivated in different climates.

In recent years, various elicitors have been applied in vitro to increase anthocyanin production in carrot tissue cultures [29–32]. However, according to our knowledge, there are no reports describing enhanced anthocyanin accumulation following elicitation in studies of carrot plants in vivo. The connection between foliar spray-application of ethephon and enhanced anthocyanin accumulation in roots has also not been reported previously. Therefore, the present study highlights a novel application of ethephon and similar ethylene-generating compounds for natural food colourant production.

### Monitoring of anthocyanins and phenolic compounds during root growth

The onset of the effect of ethephon on TMA and TPC was documented from the early stages of growth. Moreover, anthocyanin and phenolic compound levels increased for several weeks after the ethephon treatment was terminated (Figs. 3a and b). Remarkably, anthocyanins represented a higher percentage of the total phenolic compounds (Figs. 3a and b) than that previously reported in ‘Deep Purple’ carrots [2, 33]. The most outstanding difference between the TPC and TMA accumulation curves appeared at the last two harvest points, when the TPC remained unchanged, while the TMA decreased. This may indicate that non-anthocyanin phenolic compounds increased at that stage to compensate for the decreased anthocyanin accumulation.

The TMA accumulation kinetics have been shown to resemble a sigmoidal curve in some anthocyanin-rich fruits [34–36], but this pattern has not yet been demonstrated in plant roots. In this study, the kinetics of root TMA in both untreated and elicitor-treated plants could be divided into three stages: an initial stage with a rapid rate of accumulation, a transitional stage determined by a lower accumulation rate leading to a maximum concentration, and a stationary phase (Fig. 3a).

The anthocyanin profile was consistent with those previously published for ‘Deep Purple’ [2, 28, 37–39] (Fig. 4; Table 3). Overall, our analysis of the relative abundance of the five anthocyanins evaluated in both untreated and elicitor-treated carrots revealed that the increased anthocyanin content in the elicited roots was composed of an equal distribution of each of the five forms (Fig. 5a). The most outstanding difference was observed in the accumulation of cyanidin 3-xylosylgalactoside (peak 2), which was increased by up to 8 percentage points in roots of elicitor-treated plants compared to untreated plants. Nevertheless, the underlying mechanisms that determine changes in the profile of anthocyanin content during growth have not yet been determined.

### Relationship between anthocyanin and sugar contents during root growth

Several studies have highlighted the role of sugars as signalling molecules in many plant processes, including the biosynthesis of anthocyanin and, in general, of phenolic compounds [40–42]. Furthermore, a peak in sugar concentration has been shown to induce key MYB transcription factors involved in anthocyanin formation in Arabidopsis [43, 44]. Our results support these observations, since the most rapid phase of SSC and DW accumulation (from 7 to 13 weeks after sowing) was concomitant with the highest TMA and TPC accumulation rates in both untreated and treated plants (Fig. 3).

Anthocyanins undergo glycosylation, in which one or more sugar molecules are added [45]. Therefore, it can be hypothesised that the lower SSC and DW in ethephon-treated roots results from increased sugar consumption during anthocyanin biosynthesis. However, in-depth studies involving whole carrot plants are required to elucidate the dynamics of sugar metabolism in leaves and shoots.

### Patterns of anthocyanin biosynthesis-related gene expression following ethephon elicitation

The correlation between anthocyanin content and the expression of structural anthocyanin genes at different times up to 17 weeks after sowing has been reported in several carrot cultivars [23, 24]. However, this correlation in late-harvested roots has not been reported previously. From our analyses at 22 and 25-weeks after sowing (i.e. at the first and second harvest following the final ethephon treatment), we conclude that ethephon increases anthocyanin levels predominantly via upregulation of their structural genes, probably through the action of *Dc*MYB1 on *DcPAL* gene expression (Fig. 6)*.* Phenylalanine ammonia-lyase (PAL) enzymes play a key role in the biosynthesis of phenolic compounds. *DcPAL1* gene expression has been reported to be involved mainly in stress- and elicitor-induced biosynthesis of phenolic compounds, whereas *DcPAL3* is involved in anthocyanin synthesis [46, 47]. In cultured carrot cells, *Dc*EIL negatively regulates *Dc*MYB1 by binding to the *DcMYB1* promoter and suppressing its expression under non-stress conditions [48]. However, no significant variation in *DcEIL* expression was detected in the present study, indicating that degradation of the pool of *Dc*EIL protein occurs prior to *Dc*MYB1 expression.

Collectively, our results provide new insights into the kinetics of accumulation of anthocyanin forms during root growth in carrots, and suggest a priming effect of ethephon in the transcription of anthocyanin biosynthesis genes and the accumulation of anthocyanins in the root.

## Conclusions

Foliar-applied ethephon significantly enhanced anthocyanin and TPC in black carrot roots, and decreased DW and SSC. Anthocyanin levels increased for several weeks after ethephon treatment was terminated. Five cyanidin-based anthocyanin forms were identified, the relative abundance of which varied during root growth; however, there were no significant differences between untreated and ethephon-treated plants. Furthermore, the patterns of expression of biosynthetic anthocyanin genes and the *DcMYB1* transcription factor correlated with that of anthocyanin accumulation. Collectively, our findings show that ethephon treatment of ‘Deep Purple’ F1 results not only in increased acylated anthocyanin content, but also in increased anthocyanin per unit of biomass. These findings are important in devising strategies to improve key profitability parameters in food colour production.

## Additional file

Additional file 1: Table S1. Annotation, accession number and nucleotide sequences of primers to genes used for Real Time q-PCR. (DOCX 18 kb)

## Abbreviations

DFR: Dihydroflavonol 4-reductase
DW: Dry weight
EIL: EIN3-like
EIN: Ethylene insensitive
F3H: Flavanone 3-hydroxylase
FW: Fresh weight
GAE: Gallic acid equivalent
HPLC-DAD: High performance liquid chromatography-diode array detection
LC-MS/Q-TOF: Liquid chromatography coupled to (quadrupole) time-of-flight mass spectrometry
LDOX: Leucoanthocyanidin dioxygenase
MYB: myeloblastosis
PAL: Phenylalanine ammonia-lyase
SSC: Soluble solids content
TMA: Total monomeric anthocyanin content
TPC: Total phenolic content.

## References

[1] Giusti MM, Wrolstad RE (2003). Acylated anthocyanins from edible sources and their applications in food systems. Biochemical Eng J.

[2] Montilla EC, Arzaba MR, Hillebrand S, Winterhalter P (2011). Anthocyanin composition of black carrot (*Daucus carota ssp. sativus* var. *atrorubens* Alef.) cultivars Antonina, Beta Sweet, Deep Purple, and Purple Haze. J Agric Food Chem.

[3] Mazza G, Miniati E (1993). Anthocyanins in fruits, vegetables, and grains.

[4] Andersen OM, Jordheim M. The anthocyanins. In: Andersen OM, Markham KR, editors. Flavonoids: Chemistry, Biochemistry and Applications. Boca Raton, FL: CRC Press; 2005. p. 471–552.

[5] Kammerer D, Carle R, Schieber A (2004). Characterization of phenolic acids in black carrots (*Daucus carota ssp. sativus* var. *atrorubens* Alef.) by high-performance liquid chromatography/electrospray ionization mass spectrometry. Rapid Commun Mass Spectrom.

[6] Assous MTM, Abdel-Hady MM, Medany GM (2014). Evaluation of red pigment extracted from purple carrots and its utilization as antioxidant and natural food colorants. Ann Agric Sci.

[7] Iliopoulou I, Thaeron D, Baker A, Jones A, Robertson N (2015). Analysis of the Thermal Degradation of the Individual Anthocyanin Compounds of Black Carrot (*Daucus carota* L.): A New Approach Using High-Resolution Proton Nuclear Magnetic Resonance Spectroscopy. J Agric Food Chem.

[8] McCann D, Barrett A, Cooper A, Crumpler D, Dalen L, Grimshaw K (2007). Food additives and hyperactive behaviour in 3-year-old and 8/9-year-old children in the community: a randomised, double-blinded, placebo-controlled trial. Lancet.

[9] Carocho M, Barreiro MF, Morales P, Ferreira I (2014). Adding Molecules to Food, Pros and Cons: A Review on Synthetic and Natural Food Additives. Compr Rev Food Sci Food Saf.

[10] Cevallos-Casals BA, Cisneros-Zevallos L (2004). Stability of anthocyanin-based aqueous extracts of Andean purple corn and red-fleshed sweet potato compared to synthetic and natural colorants. Food Chem.

[11] Kirca A, Özkan M, Cemeroglu B (2006). Stability of black carrot anthocyanins in various fruit juices and nectars. Food Chem.

[12] Lapidot T, Harel S, Akiri B, Granit R, Kanner J (1999). pH-Dependent forms of red wine anthocyanins as antioxidants. J Agric Food Chem.

[13] Delgado-Vargas F, Paredes-Lopez O (2003). Natural Colorants for Food and Nutraceutical Uses.

[14] Galvano F (2005). The chemistry of anthocyanins. Functional ingredients magazine.

[15] Tanaka Y, Sasaki N, Ohmiya A (2008). Biosynthesis of plant pigments: anthocyanins, betalains and carotenoids. Plant J.

[16] Ali MB, Yu KW, Hahn EJ, Paek KY (2006). Methyl jasmonate and salicylic acid elicitation induces ginsenoside accumulation, enzymatic and non-enzymatic antioxidant in suspension culture of *Panax ginseng* roots in bioreactors. Plant Cell Rep.

[17] Ahlawata S, Saxena P, Alam P, Wajid S, Abdin MZ (2014). Modulation of artemisinin biosynthesis by elicitors, inhibitor, and precursor in hairy root cultures of *Artemisia annua* L. J Plant Interact.

[18] Holton TA, Cornish EC (1995). Genetics and Biochemistry of Anthocyanin Biosynthesis. Plant Cell.

[19] Lalusin AG, Nishita K, Kim SH, Ohta M, Fujimura T (2006). A new MADS-box gene (*IbMADS10*) from sweet potato (*Ipomoea batatas* (L.) Lam) is involved in the accumulation of anthocyanin. Mol Gen Genomics.

[20] Lu QN, Yang Q (2006). cDNA cloning and expression of anthocyanin biosynthetic genes in wild potato (*Solanum pinnatisectum*). Afr J Biotechnol.

[21] Mano H, Ogasawara F, Sato K, Higo H, Minobe Y (2007). Isolation of a Regulatory Gene of Anthocyanin Biosynthesis in Tuberous Roots of Purple-Fleshed Sweet Potato. Plant Physiol.

[22] Hirner AA, Veit S, Seitz HU (2001). Regulation of anthocyanin biosynthesis in UV-A-irradiated cell cultures of carrot and in organs of intact carrot plants. Plant Sci.

[23] Yildiz M, Willis DK, Cavagnaro PF, Iorizzo M, Abak K, Simon PW (2013). Expression and mapping of anthocyanin biosynthesis genes in carrot. Theor Appl Genet.

[24] Xu ZS, Huang Y, Wang F, Song X, Wang GL, Xiong AS (2014). Transcript profiling of structural genes involved in cyanidin-based anthocyanin biosynthesis between purple and non-purple carrot (*Daucus carota* L.) cultivars reveals distinct patterns. BMC Plant Biol.

[25] Singleton VL, Rossi JA (1965). Colorimetry of total phenolics with phosphomolybdic–phosphotungstic acid reagents. Am J Enol Viticult.

[26] Stranathan M, Hastings C, Trinh H, Zimmerman JL (1989). Molecular evolution of two actin genes from carrot. Plant Mol Biol.

[27] Lütken H, Jensen LS, Topp SH, Mibus H, Müller R, Rasmussen SK (2010). Production of compact plants by overexpression of *AtSHI* in the ornamental Kalanchoë. Plant Biotechnol J.

[28] Kammerer D, Carle R, Schieber A (2003). Detection of peonidin and pelargonidin glycosides in black carrots (*Daucus carota ssp. sativus* var. *atrorubens* Alef.) by high-performance liquid chromatography/electrospray ionization mass spectrometry. Rapid Commun Mass Spectrom.

[29] Rajendran L, Ravishankar GA, Venkataraman LV, Prathiba KR (1992). Anthocyanin production in callus cultures of *Daucus carota* as influenced by nutrient stress and osmoticum. Biotechnol Lett.

[30] Rajendran L, Suvarnalatha G, Ravishankar GA, Venkataraman LV (1994). Enhancement of anthocyanin production in callus cultures of *Daucus carota* L. under the influence of fungal elicitors. Appl Microbiol Biotechnol.

[31] Sudha G, Ravishankar GA (2003). Elicitation of anthocyanin production in callus cultures of *Daucus carota* and the involvement of methyl jasmonate. Acta Physiol Plant.

[32] Suvarnalatha G, Rajendran L, Ravishankar GA, Venkataraman LV (1994). Elicitation of anthocyanin production in cell cultures of carrot (*Daucus carota* L) by using elicitors and abiotic stress. Biotechnol Lett.

[33] Leja M, Kamińska I, Kramer M, Maksylewicz-Kaul A, Kammerer D, Carle R (2013). The content of phenolic compounds and radical scavenging activity varies with carrot origin and root color. Plant Foods Hum Nutr.

[34] Ribereau-Gayon P, Markakis P (1982). The anthocyanins of grapes and wines. Anthocyanins as Food Colors.

[35] Usenik V, Štampar F, Veberic R (2009). Anthocyanins and fruit colour in plums (*Prunus domestica* L.) during ripening. Food Chem.

[36] Rogez H, Pompeu DR, Akwie SNT, Larondelle Y (2011). Sigmoidal kinetics of anthocyanin accumulation during fruit ripening: A comparison between açai fruits (*Euterpe oleracea*) and other anthocyanin-rich fruits. J Food Comp Anal..

[37] Netzel M, Netzel G, Kammerer D, Schieber A, Carle R, Simons L (2007). Cancer cell antiproliferation activity and metabolism of black carrot anthocyanins. Innov Food Sci Emerg Technol.

[38] Türkyılmaz M, Yemiş O, Özkan M (2012). Clarification and pasteurisation effects on monomeric anthocyanins and percent polymeric colour of black carrot (*Daucus carota* L.). Food Chem.

[39] Algarra M, Fernandes A, Mateus N, de Freitas V (2014). Esteves da Silva JCG, Casado J. Anthocyanin profile and antioxidant capacity of black carrots (*Daucus carota* L. ssp. sativus var. atrorubens Alef.) from Cuevas Bajas, Spain. J Food Comp Anal.

[40] Mita S, Murano N, Akaike M, Nakamura K (1997). Mutants of *Arabidopsis thaliana* with pleiotropic effects on the expression of the gene for beta-amylase and on the accumulation of anthocyanin that are inducible by sugars. Plant J.

[41] Baier M, Hemmann G, Holman R, Corke F, Card R, Smith C (2004). Characterization of mutants in Arabidopsis showing increased sugar-specific gene expression, growth, and developmental responses. Plant Physiol.

[42] Rolland F, Baena-González E, Sheen J (2006). Sugar sensing and signaling in plants: conserved and novel mechanisms. Annu Rev Plant Biol.

[43] Lloyd JC, Zakhleniuk OV (2004). Responses of primary and secondary metabolism to sugar accumulation revealed by microarray expression analysis of the Arabidopsis mutant, *pho3*. J Exp Bot.

[44] Teng S, Keurentjes J, Bentsink L, Koornneef M, Smeekens S (2005). Sucrose-specific induction of anthocyanin biosynthesis in Arabidopsis requires the *MYB75*/*PAP1* gene. Plant Physiol.

[45] González-SanJosé ML, Diez C (1992). Relationship between anthocyanins and sugars during the ripening of grape berries. Food Chem.

[46] Ozeki Y, Matui M, Sakuta M, Matsuoka M, Ohasi Y, Kano-Murakami Y (1990). Differential regulation of phenylalanine ammonia-lyase genes during anthocyanin synthesis and by transfer effect in carrot cell suspension cultures. Physiol Plant.

[47] Maeda K, Kimura S, Demura T, Takeda J, Ozeki Y (2005). DcMYB1 acts as a transcriptional activator of the carrot phenylalanine ammonia-lyase gene (*DcPAL1*) in response to elicitor treatment, UV-B irradiation and the dilution effect. Plant Mol Biol.

[48] Miyahara T, Satoh S, Maeda K, Kimura S, Sasaki N, Ozeki Y (2010). Isolation of *Daucus carota* ethylene insensitive3-like (*DcEIL*) involved in stress-inducible *DcMYB1* expression in suspension-cultured carrot cells. Plant Biotechnol.

