# Supplementary material for: Foliar-applied ethephon enhances the content of anthocyanin of black carrot roots (Daucus carota ssp. sativus var. atrorubens Alef.)
Source: BMC Plant Biol. 2017 Apr 4;17:70. doi: 10.1186/s12870-017-1021-7 (PMC5381149; doi:10.1186/s12870-017-1021-7)
Supplement: Additional file 1: Table S1. — Annotation, accession number and nucleotide sequences of primers to genes used for Real Time q-PCR. (DOCX 18 kb) [file 12870_2017_1021_MOESM1_ESM.docx]

Additional file 1: Table S1. Annotation, accession number and nucleotide sequences of primers to genes used for Real Time q-PCR.

| Gene family | Annotation | GenBank ID | Forward primer 5′-3′ | Reverse primer 5′-3′ |
| --- | --- | --- | --- | --- |
| *Actin* | *Actin2* | X17525.1 | attgggaatggagtctgctg | gatcgatcctccaatccaga |
| *EIN3* | *EIL* | AB525913.1 | ggagcctctgacaatcttcg | agcagacaacagggagccta |
| *MYB* | *MYB1* | FJ478180.1 | tcggtcacagactcatcagc | tgcaccaaaagtccatttca |
| *PAL* | *PAL1* | D85850.1 | gcaagggctgtggtagagag | ctctcaagacacgccaacaa |
|  | *PAL3* | AB089813.1 | CTGGCATGGCCTCTATGGTACT | TGTCCTGGATGGTGCTTCAACT |
| *F3H* | *F3H1* | AF184270.1 | GAGTACAGTGAGAAGCTGATGGGTC | GGTTGAGGGCACTTGGGATAG |
| *DFR* | *DFR1* | AF184271.1 | GTTATCAAGCCTACCGTACAGGG | AGTTCCAGCAGACGAAGTGTAAAT |
| *LDOX* | *LDOX2* | AF184274.1 | AGGTGCCCACAGTCGACATAGC | CGCCTGTCCAGCCACTCTAA |

Gene abbreviations: *EIN*, *Ethylene insensitive*; *MYB*, *myeloblastosis*; *PAL*, *Phenylalanine ammonia-lyase*; *F3H*, *Flavanone 3-hydroxylase*; *DFR*, *Dihydroflavonol 4-reductase*; *LDOX, Leucoanthocyanidin dioxygenase*. Primers for *PAL3*, *F3H1*, *DFR1* and *LDOX2* genes were reported earlier (Xu et al. 20014). Primers for *Actin2*, *EIN3*, *MYB1* and *PAL1* genes were designed using Primer3 online software (<http://bioinfo.ut.ee/primer3-0.4.0/primer3/>).
